# Perineal lipoma associated with penoscrotal transposition in a neonate

**DOI:** 10.4103/0971-9261.44772

**Published:** 2008

**Authors:** Anup Mohta, Swarup Das, Mamta Sengar

**Affiliations:** Department of Pediatric Surgery, Chacha Nehru Bal Chikitsalaya and Maulana Azad Medical College, Geeta Colony, Delhi-110 031, India

**Keywords:** Lipoma, neonate, penoscrotal transposition

## Abstract

A neonate with perineal lipoma associated with penoscrotal transposition and bifid scrotum is reported.

## INTRODUCTION

Perineal lipoma is uncommon in neonates and its association with penoscrotal transposition is even more uncommon.

## CASE REPORT

**Figure 1:**
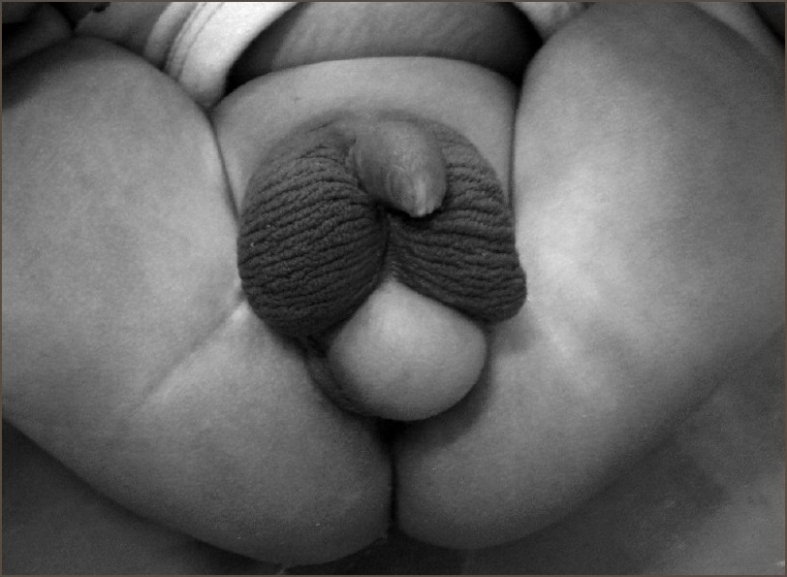
Clinical photograph showing penoscrotal transposition, bifid scrotum, and perineal lipoma

A 2-week-old baby presented with a mass beneath the scrotum. There was no difficulty in passing urine. On examination, the penile shaft was placed between two halves of the scrotum embedded along the scrotum and the posterior aspect of the scrotum was partially separated. A 3–4 cm midline perineal mass was present just beneath the divided scrotum; it was covered with smooth shiny skin without any rugosity and was soft in consistency [Figure 1]. This was diagnosed as perineal lipoma. Both testicles were palpable in the scrotum. The urethral meatus was present at the tip of the glans. The anorectal examination was normal. Complete blood count and urine examination were normal. The ultrasound of the urinary tract did not reveal any anomaly.

The child underwent scrotoplasty using bilateral V-Y plasty and excision of the perineal mass at five months of age. The postoperative cosmetic result was satisfactory to the parents. Histological examination of the excised mass showed mature fat cells without atypical cells and absence of any muscle fibers in the subcutaneous region.

## DISCUSSION

Developmental scrotal anomalies are uncommon occurrences. Bifid scrotum and penoscrotal transposition are commonly seen, while accessory or ectopic scrotum is very rare.[1] Perineal lipomas are said to occur infrequently in association with scrotal anomalies and are rarely reported in neonates.[1–3] They are more commonly seen to occur with anorectal malformations.[4]

At the fourth week of gestation, a genital swelling normally appears at both sides of the inguinal region and gradually forms the labioscrotal swelling at 10–12 weeks of gestation. These swellings migrate to the caudal portions and merge beneath the penis, which remains as the scrotal raphe, the line of fusion. It has been suggested that scrotal anomalies may result from early division and/or abnormal migration of the labioscrotal swelling.[5] Unilateral failure or abnormal migration might result in unilateral penoscrotal transposition or ectopic scrotum, and early division of a labioscrotal swelling with subsequent abnormal migration might result in an accessory scrotum. Park *et al*[1] suggested that the concomitant development of a perineal lipoma might interrupt the normal migration of the labioscrotal swelling leading to development of bifid scrotum and penoscrotal transposition. They classified the perineal lipomas into two types. The protruding type that has a disruptive effect on the continuity of the developing caudal labioscrotal swelling; and the peduncular type, an outpouching single mass composed of two types of tissue elements, which originates by early division of pleuripotential labioscrotal tissue elements. These need to be differentiated from accessory scrotum, sacrococcygeal teratoma, perineal hernia, or other perineal tumors.[2] A perineal lipoma can be differentiated from accessory scrotum by absence of rugosity on the skin during clinical examination and smooth muscle (dartos) fibers on histological examination. Radiological investigations like ultrasonography and magnetic resonance imaging may be necessary to differentiate perineal lipoma from other lesions in the region.

Perineal lipoma that occurs in association with the scrotal anomalies alone can be excised without difficulty along with scrotal reconstruction. The perineal lipoma that occurs in association with the anorectal malformation can be excised at the time of definitive procedure for the anorectal anomaly.
